# The Impact of Electronic Health Record Interoperability on Safety and Quality of Care in High-Income Countries: Systematic Review

**DOI:** 10.2196/38144

**Published:** 2022-09-15

**Authors:** Edmond Li, Jonathan Clarke, Hutan Ashrafian, Ara Darzi, Ana Luisa Neves

**Affiliations:** 1 National Institute for Health and Care Research (NIHR) Imperial Patient Safety Translational Research Centre Institute of Global Health Innovation Imperial College London London United Kingdom

**Keywords:** electronic health records, interoperability, patient safety, systematic literature review, health information exchange, digital health

## Abstract

**Background:**

Electronic health records (EHRs) and poor system interoperability are well-known issues in the use of health information technologies in most high-income countries worldwide. Despite the abundance of literature exploring their relationship, their practical implications on patient safety and quality of care remain unclear.

**Objective:**

This study aimed to examine how EHR interoperability affects patient safety, or other dimensions of care quality, in high-income health care settings.

**Methods:**

A systematic search was conducted using 4 web-based medical journal repositories and grey literature sources. The publications included were published in English between 2010 and 2022, pertaining to EHR use, interoperability, and patient safety or care quality in high-income settings. Screening was completed by 3 researchers in accordance with the PRISMA (Preferred Reporting Items for Systematic Reviews and Meta-Analyses) guidelines. Risk of bias assessments were performed using the Risk of Bias in Non-randomized Studies of Interventions and the Cochrane Risk of Bias 2 tools. The findings were presented as a narrative synthesis and mapped based on the Institute of Medicine’s framework for health care quality.

**Results:**

A total of 12 studies met the inclusion criteria to be included in our review. The findings were categorized into 6 common outcome measure categories: patient safety events, medication safety, data accuracy and errors, care effectiveness, productivity, and cost savings. EHR interoperability positively influenced medication safety, reduced patient safety events, and reduced costs. Improvements in time saving and clinical workflow are mixed. However, true measures of effect are difficult to determine with certainty because of the heterogeneity in the outcome measures used and notable variation in study quality.

**Conclusions:**

The benefits of EHR interoperability on the quality and safety of care remain unclear and reflect extensive heterogeneity in the interventions, designs, and outcome measures used. The establishment of common health information technology research outcome measures would support higher-quality research on the topic. Future research efforts should focus on both the positive and negative impacts of interoperable EHR interventions and explore patient perspectives, given the growing trend for patient involvement and stewardship over their own electronic clinical data.

**Trial Registration:**

PROSPERO CRD42020209285; https://www.crd.york.ac.uk/prospero/display_record.php?RecordID=209285

**International Registered Report Identifier (IRRID):**

RR2-10.1136/bmjopen-2020-044941

## Introduction

### Background

Electronic health records (EHRs) have become a mainstay digital solution for high-income health systems globally [1-3]. Despite the growing integration of such technologies into the routine workflows of health care providers, sizable challenges remain that prevent EHRs from fulfilling their full potential. One such hurdle is the lack of interoperability.

The Healthcare Information and Management Systems Society defines interoperability as “the ability of different information systems, devices and applications (systems) to access, exchange, integrate and cooperatively use data in a coordinated manner, within and across organizational, regional and national boundaries, to provide timely and seamless portability of information and optimize the health of individuals and populations globally” [4]. However, this may have drastically different implications depending on one’s role and perspective in a health system.

From a technological standpoint, EHR interoperability can be defined as *“*the ability of two or more applications to communicate effectively without compromising the content of the transmitted EHR*”* [5]. However, barriers such as hardware, syntax, and system usability often hinder the implementation of this vision [6-8]. The adoption of common standards in terminology, content, and security has been proposed to facilitate different levels of interoperability within or across health care settings [4,9].

From a public health, administrative, or policy-making perspective, EHR interoperability may entail *“*electronic health information that is shared appropriately between healthcare and public health partners in the right format, through the right channel at the right time*”* [10]. For end users such as health care providers and patients, the notion of interoperability often focuses more on the practical functionalities of EHR interoperability. For health care providers, this may include being able to remotely access care records from another health care setting, electronically correspond with other providers, and coordinate complex care plans with external health care organizations [2,3,11-13]. For patients, interoperability may mean a more seamless experience when seeking care from various health care providers or feeling an increased sense of empowerment by having greater access to their health records [14].

Lack of interoperability may negatively impact all 6 dimensions of quality of care outlined by the Institute of Medicine (IoM) framework: safety, effectiveness, patient-centeredness, timeliness, efficiency, and equity [15-17]. These shortcomings may range from inaccurate or fragmented patient health records across multiple care providers and delayed communication between care teams to increased costs resulting from duplicated efforts by staff and health care resources used [7,18-20]. The introduction of EHRs has been attributed to benefits such as improving health care worker productivity, facilitating public health disease surveillance and research, and even generating cost savings [21]. While the implementation of EHR interoperability was speculated to realize further benefits, such as improved care coordination and additional potential savings, current evidence remains mixed [3,8,22].

Although the issues associated with EHR interoperability are well recognized in high-income health care settings [8], evidence exploring its impact on the 6 dimensions of care quality remains relatively scarce. Nearly 2 decades after the initial adoption of EHR in high-income countries (HICs) such as the United States and the United Kingdom [8,22-24], there is a need to revisit and review the currently available literature to evaluate EHR interoperability, its impact on quality of care, and particularly in terms of patient safety.

### Aims

This systematic review aims to evaluate the impact of EHR interoperability on the IoM’s 6 domains of health care quality in HICs [15].

## Methods

### Search Strategy

A literature search was conducted for publications published between 2010 and 2020 on 4 databases (PubMed, MEDLINE, Embase, and PsycINFO). Publications from grey literature sources and relevant papers identified from the references of the screened articles were also included. A more thorough description of the search strategy and inclusion criteria was previously published as a study protocol [25].

We performed an additional search for publications published between March 2020 and June 2022. As the onset of COVID-19 has had profound implications on many aspects of health care technologies and policies, this supplementary search was conducted to account for any new studies published during that period. Otherwise, there were no further deviations from the methods described in the previously published protocol.

### Study Selection Criteria

This systematic review included studies fulfilling the following criteria: (1) studies took place in HICs as defined by the World Bank where *“*the gross national income (GNI) *per capita* is higher than $13,205 USD*”* [26,27], (2) investigated EHRs or other health information technologies (HITs) that facilitate the sharing of clinical information between health care providers, and (3) contained outcomes concerning patient safety or quality of care. Only the studies published in English were included. No other filters, such as the study design, type, or publication country of origin, were used. Screening and selection of publications were performed by a total of 3 reviewers. Two reviewers initially independently screened the body of articles derived from the database searches to be considered for inclusion. This was performed iteratively at the title, abstract, and full-text levels in accordance with the PRISMA (Preferred Reporting Items for Systematic Reviews and Meta-Analyses) diagram [28]. Cohen κ statistic was used to measure interrater reliability [29]. Discrepancies in article selection were arbitrated by a third reviewer.

### Data Extraction

Data extraction was completed by the first reviewer using a standardized Microsoft Excel spreadsheet. The content was then reviewed by the other 2 reviewers to ensure data quality and consistency. The characteristics and data extracted from each study included the name of the authors, year of publication, study design, study setting, study population and size, outcome measures, and general findings.

### Risk of Bias and Quality Assessment

The Cochrane Risk of Bias Tool was used to assess randomized control trials for bias, whereas the “Risk of Bias in Non-Randomized Studies—of Interventions” tool was used for nonrandomized trials [30]. Risk of bias assessments were conducted by 2 reviewers and any disagreements were resolved by a third investigator.

### Data Synthesis

A narrative synthesis of the findings was conducted. Relevant findings and outcome measures were grouped into subcategories and organized based on the 6 domains found in the IoM health care quality framework. Given the variety of outcome measures used in the included studies, a meta-analysis was not performed.

## Results

### Overview

**Figure 1:**
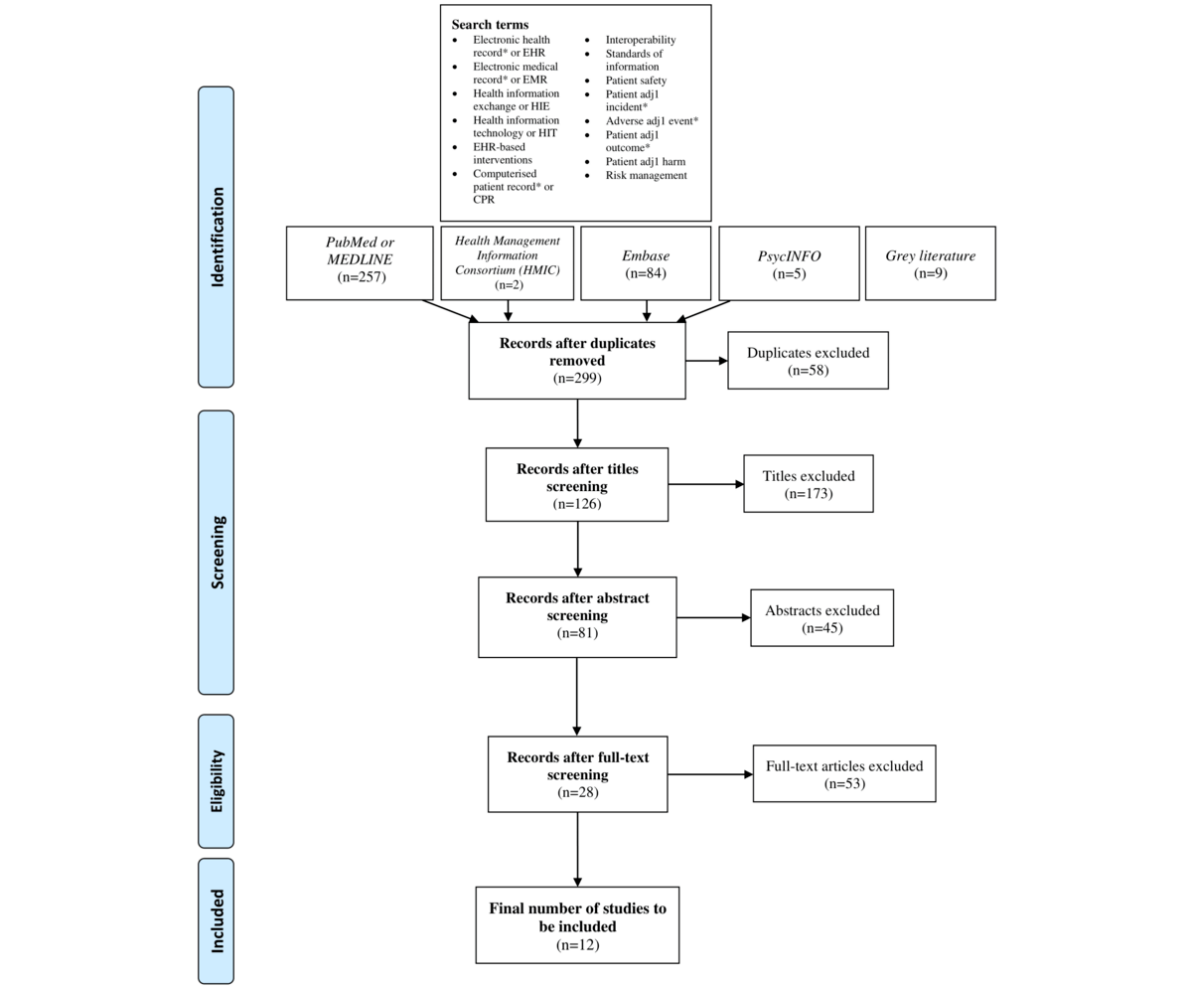
PRISMA (Preferred Reporting Items for Systematic Reviews and Meta-Analyses) systematic review search strategy and screening process flow diagram. Search terms appear as used in Li et al [25].

The initial search using computerized databases yielded 299 publications (Figure 1). After screening the titles, 173 publications were excluded as they did not meet the inclusion criteria. Upon further screening of the abstracts, 45 articles were rejected because they were either not relevant or were not studies, but rather commentaries or opinion pieces. Following full-text screening and agreement among the 3 reviewers, 53 publications were excluded because they did not satisfy all the requirements of the PICO (population, intervention, control, and outcomes) inclusion criteria.

A final total of 12 papers were selected for this systematic review.

κ statistic for full-text screening was 0.52, indicating fair agreement [29].

### Description of Included Studies

The 12 selected studies were published between 2013 and 2020. The included studies were predominantly nonrandomized trials, including observational studies (n=5) [31-35], cross-sectional studies (n=2) [36,37], retrospective analyses of patient safety reports (n=2) [38,39], case studies (n=1) [40], and a simulation study (n=1) [41]. Only one randomized controlled trial was included [42]. Further details on the included studies have been provided in Table 1.

**Table 1:** Summary characteristics of included studies.

Study	Publication year	Journal	Study type	Stated aims or objectives	Date or duration of intervention	Study population or settings
Reed et al [34]	2020	*American Journal of Managed Care*	Observational study	To examine whether providers’ timely access to clinical information through shared inpatient-outpatient EHRsa was associated with follow-up visits, return emergency department visits, or readmissions after hospital discharge in patients with diabetes.	2005-2011	241,510 hospitalized patients with diabetes discharged home from 17 hospitals where a new inpatient EHR system is being gradually introduced which integrates with an existing outpatient EHR system.
Wong et al [35]	2020	*Journal of the American Medical Directors Association*	Observational study	To assess the impact of implementing a new electronic medical records transfer mechanisms or process to improve the transfer of medical records when transitioning patients between nursing facilities and acute settings	2020	HOPE^b^ SNF^c^ Collaborative of 25 nursing facilities working with 3 hospitals in a local health network.
Howe et al [38]	2018	*JAMA*	Retrospective analysis of patient safety reports	To explore how EHR usability can contribute to patient harm by reviewing patient safety reports from the Pennsylvania Patient Safety Authority database.	2013-2016	Patient safety reports from the Pennsylvania Patient Safety Authority database derived from 571 health care facilities.
Biltoft et al [40]	2018	*American Journal of Health-System Pharmacy*	Case study	To improve IV^d^ infusion: medication safety accuracy, timeliness, and efficiency of IV medication documentation Free up pharmacist and nurse time for direct patient care Increase revenue by improving reimbursement for IV medications in outpatient areas	October 2013, lasting for 7 months	Regional health system consisting of 8 hospitals, excludes NICUs^e^
D’Amore et al [36]	2018	*AMIA^f^symposium*	Cross-sectional study	To examine testing artifacts from recent certification through automated tooling and manual review to identify compatibility and usability issues.	January 2018	854 C-CDA^g^ documents were selected from the Office of the National Coordinator for Health Information Technology publicly available repository. After screening for duplicates, invalid XML, and documents not confirming to C-CDA 2.1 standards, 401 C-CDA documents were examined
Adams et al [39]	2017	*Applied Clinical Informatics*	Retrospective analysis of patient safety reports	Overall study was to understand patient safety consequences resultant from interoperability issues between EHRs and HIT^h^. Specific objectives were: To identify patient safety incident reports that reflect EHR interoperability challenges with other health IT. To perform a detailed analysis of these reports to understand the health IT systems involved, the clinical care processes impacted, whether the incident occurred within or between provider organizations, and the reported severity of the patient safety events.	2009-2016	1.735 million PSE^i^ reports from the Pennsylvania Patient Safety Authority’s Pennsylvania Patient Safety Reporting System, attained through the ISMP^j^, and a large health care system in the Mid-Atlantic United States; 209 (8%) PSE reports of the 2625 health IT reports were determined to be related to interoperability between the EHR and another health IT system.
Elysee et al [31]	2017	*Medicine (United States)*	Observational study	To empirically examine how the 3 capabilities (HIE^k^, interoperability, medication reconciliation) influence one another so the appropriate policy can be applied where it can have the greatest impact.	2013	AHA^l^ Annual IT Survey responses; 1330 hospitals were included. 2013 AHA Annual Survey IT Supplement database to obtain a nationally representative sample of nonfederal acute care hospitals that (1) include acute care general medical and surgical, general children’s, and cancer hospitals (2) use any type of electronic exchange or sharing of care summaries with other providers
Motulsky et al [32]	2016	*Studies in Health Technology and Informatics*	Observational study	Evaluated the accuracy and usability of SQIM software for documenting the list of current medications for patients at admission to hospital and comparing with medication lists with pharmacies via fax.	June 2014 to January 2015	111 patients, average age of 76 years, 51% female, average of 11 medications. On the basis of tertiary care center in Montreal, Canada
Akbarov et al [37]	2015	*Drug Safety*	Cross-sectional study	To investigate the feasibility of linked primary and secondary care EHR data for surveillance of medication safety. Objectives included assessing the prevalence of 22 medication safety indicators, investigating associations with patient and practice characteristics, and investigating variation between general practices.	April 2012	52 general practices affiliated with 205,519 patients in Salford, United Kingdom
Munck et al [42]	2014	*Danish Medical Journal*	Randomized control trial+Likert scale questionnaire	Examines time expenditure and impact on workflow the use of an integrated shared medical record has on medication reconciliation at hospital admissions	June 2010	Sixty-two patient consultations, 18 physicians participated from the accident and emergency department at Køge Hospital—a university-affiliated hospital.
Koldby et al [41]	2013	*Studies in Health Technology and Informatics*	Simulation study	To evaluate how integration between digital dictation and EHRs impacts workflow, and functionality, and identify areas requiring further improvement.	N/A^m^	Three doctors (2 surgeons, one pediatrician) and 3 medical secretaries, Herlev Hospital in Copenhagen, Denmark
Lee et al [33]	2013	*Studies in Health Technology and Informatics*	Observational study	To develop and implement a workflow-based multidisciplinary hand-over information system, integrated with medical record browsing, multidisciplinary hand-over, and event tracking to improve the correctness and effectiveness of communication among the medical team members.	2 years, auditing was completed every 3 months	40+ seed anchors were trained on the use of the cross-disciplinary team hand-over information system. They were responsible for training nurses in their respective wards; no further detail on sample size

^a^EHR: electronic health record.

^b^HOPE: Health Optimization for Elders.

^c^SNF: Skilled Nursing Facility.

^d^IV: intravenous.

^e^NICU: neonatal intensive care unit.

^f^AMIA: American Medical Informatics Association.

^g^C-CDA: consolidated clinical document architecture.

^h^HIT: health information technology.

^i^PSE: patient safety event.

^j^ISMP: Institute for Safe Medication Practices.

^k^HIE: health information exchange.

^l^AHA: American Hospital Association.

^m^N/A: not applicable.

### Description of Interventions in Included Studies

The intervention of interest examined in this systematic review is the implementation of interventions that intend to improve EHR interoperability with other EHRs or health IT systems. The review incorporated interventions that aimed to reduce interoperability errors, inaccurate patient records with inappropriate units of measurement, incorrect medication doses, and omission of codes or units of measure in laboratory results [36,43].

Other studies included in this review encompassed interventions that enabled interoperability between hospital-based EHRs, primary care databases, as well as medical devices such as infusion pumps [40]. Across the reviewed studies, interoperability ranged from bidirectional reading and writing of data to unidirectional writing of clinical information to a medical device or record.

### Outcomes

#### Summary of Outcome Measures

The outcomes explored by the 12 studies varied considerably and could be broadly grouped into 6 categories belonging to 3 main domains found in the IoM health care quality framework: (1) patient safety events (PSEs); (2) medication safety; (3) data sharing, accuracy, and errors; (4) care effectiveness; (5) productivity; and (6) cost savings. As all outcome subtypes could be categorized into 3 out of the 6 domains (ie, safety, effectiveness, and efficiency), only these 3 domains are included in our review for clarity. It should be noted that medication safety typically would be classified as a part of patient safety. However, as it is a common outcome specifically evaluated across many of the included studies, it is considered an independent outcome of interest in this review. Table 2 provides an overview of the outcome measures featured in each study. A detailed summary of the main findings is presented in Multimedia Appendix 1 [31-42].

**Table 2:** Outcome measures explored by the included studies, mapped onto the Institute of Medicine health care quality framework [15].

	Safety				Effectiveness		Efficiency	
Study, year	Patient safety events	Medication safety	Data sharing, accuracy, and errors	Care effectiveness		Productivity		Cost savings
Reed et al [34], 2020	✓^a^			✓				
Wong et al [35], 2020			✓					
Howe et al [38], 2018	✓	✓						
Biltoft et al [40], 2018	✓	✓	✓					✓
D’Amore et al [36], 2018		✓	✓					
Adams et al [39], 2017	✓	✓						
Elysee et al [31], 2017			✓					
Motulsky et al [32], 2016		✓	✓					
Akbarov et al [37], 2015		✓						
Munck et al [42], 2016		✓				✓		
Koldby et al [41], 2013	✓					✓		
Lee et al [33], 2013	✓					✓		

^a^✓: denotes that the specified outcome measure was present and explored in the study.

#### Patient Safety Events

A total of 6 studies included PSEs as outcome measures [33,38-41]. In 2 studies that reviewed patient records, EHR interoperability was responsible for only a small minority of safety events. Howe et al [38] found that 18.1% (102/557) of EHR-related PSEs were specifically attributed to interoperability issues. Similar results were reported by Adams et al [39], where 7.9% (209/2625) of patient safety incidents were related to problems with EHR interoperability. Notably, however, most of the problems that resulted in safety events that reached patients did not cause any direct harm (111/209, 53.1%) [39]. EHR interoperability issues resulting in medication (42/209, 20%), laboratory (33/209, 15.7%), or radiology-related (22/209, 10.5%) events comprised the largest categories of safety incidents identified [39]. These PSEs were more common when sharing clinical information among different EHR systems within a health care facility rather than when communicating with other health care providers externally [39].

The study by Reed et al [34] also examined the rates of adverse clinical events in patients with diabetes 30 days after discharge from the hospital using a new shared, integrated EHR. Adverse clinical events were determined using emergency department visits and hospital readmissions as proxy measures. However, the study found no significant change in the rates of emergency department visits (16.4% vs 16.7%) or readmissions (9.5% vs 9.4%) [34].

#### Medication Safety

Medication-related measures are some of the most used outcome measures for assessing the impact of EHR interoperability on patient safety.

For tasks such as medication reconciliation, Munck et al [42] evaluated the impact of emergency department clinicians using an EHR system interoperable with national shared medication records, compared with a standalone EHR system. Participating clinicians reported “unambiguous support” of shared medication record integration due to their perceived utility and ease with which it can be incorporated with little detriment to their workflow [42]. The clinician workload was not perceived to be different between interoperable EHRs and standalone systems [42]. In contrast, improvements in the accuracy of medication lists and communication between health care providers and patients have not been observed [42].

An observational study by Motulsky et al [32] investigated the accuracy of a new digital application introduced for documenting medications when patients present to the hospital by integrating data derived from various points along a patient’s encounter with the health care system from prescription to medication review. This was compared against a nonintegrated list from a community pharmacy. Approximately 64% of patients had discrepancies in their hospital’s medication lists, which were categorized into 3 main types of errors: (1) false positive, that is, medications listed that should not have been present, (2) false negative, that is, medications not found on the list but should have been present, and (3) duplication of medications. Of the 111 participants, 442 discrepancies were reported in their medication lists: (1) 44.6% had medications on the hospital-based list that should not have been, (2) 43.9% had current medications missing from hospital-based lists that should have been present, and (3) 11.5% contained duplicates [32].

Findings from Howe et al [38] highlighted that medication-administration safety events accounted for 37% (n=207) of the 557 EHR-related patient safety reports of events that reached patients [38]. This categorization included adverse drug events and incorrect medication dosing or route of administration [38].

Interoperability between EHR systems and other medical devices, such as infusion pumps, has also been found to be beneficial for patient safety. In the study by Biltoft et al [40], the authors noted that the implementation of an interoperable smart pump-EHR program resulted in an average number of alerts that reportedly decreased by 22% (n=1845 vs n=1447) monthly. The corresponding number of infusions requiring intervention by health care staff also dropped by nearly 20% (n=119 vs n=96), in addition to an annual reduction of staff-reported safety events from four to one [40]. By using smart pumps to prepopulate infusion parameters based on clinical data retrieved directly from interoperable EHRs, approximately 3.5 million data entry keystrokes and opportunities for errors across 8 participating hospitals were avoided monthly [40]. Clinicians have also identified an additional benefit of being able to adjust intravenous medication administration in response to a patient’s changing clinical parameters accessible directly from the EHR [40].

Finally, Elysee et al [31] examined the relationship between hospitals implementing health information exchanges (HIEs), interoperability, and medication reconciliation [31]. For successful adoption and use of HIEs, clinical information-sharing functionality is not only needed between various secondary or community-based health care facilities but also with patients. The authors concluded that these 3 capabilities are closely linked and that stalling the implementation of one of these elements in hospitals would have a detrimental impact on the adoption of the other two.

#### Data Sharing, Accuracy, and Errors

The relationship between EHR interoperability and its impact on data accuracy, sharing, and errors was explored in 5 studies.

Taking a more longitudinal perspective, D’Amore et al [36] investigated how data quality in EHRs changed in American Veterans Affairs (VA) hospitals through the increasing use of systems with greater interoperability. Using 3 independent evaluation tools, the authors found a general increase in the scope and accuracy of the clinical data being shared [36]. With HL7 Schematron testing, 86.3% (346/401) of electronic health documents contained 1695 errors, averaging 4.9 errors per clinical document [36]. Finally, using a data quality algorithm, 21,304 alerts (indicating issues in either the completeness or syntax of the record) were generated from the 401 documents examined, averaging 53.1 alerts per document [36]. 57% of these alerts were triggered due to issues surrounding data completeness, and 43% from syntax [36].

Compared with prior research, D’Amore et al [44] highlighted that the federal program for HIT certification has resulted in notable developments in the scope of information included in consolidated clinical document architecture documentation [44]. An example provided was that of implanted devices, a category not previously included in prior consolidated clinical document architecture versions [36,44].

Biltoft et al [40] reported that the rates of appropriate or correct patient ID entries across 8 participating hospitals increased from 35.5% to 81% as a result of the information being automatically filled in from interoperable EHRs when compared with the clinicians manually entering patient details [40]. Similarly, the authors also indicated a 22% (1845-1447) reduction in the average monthly infusion pump alerts as well as a 19% (119-96) reduction in the number of errors that necessitated reprogramming of the infusion pump [40]. Finally, the authors also observed a 33% (166-111) decline in the mean number of cancelled infusions per month with the introduction of EHRs interoperable with smart pumps for medication infusion [40].

A more recent study by Wong et al [35]*,* performed during the initial months of the COVID-19 pandemic, explored the introduction of a novel workflow process using software to integrate outpatient e-fax data inputs to inpatient EHRs to improve data sharing between transitions of care and limit disease transmission. Nine weeks after debuting their new process, the authors reported that it was utilized in 287 instances across the three-hospital system, with uptake and use trending positively [35]. Feedback from hospital staff was also largely positive, with only minor instances of data entry errors such as the system not handling double-sided documents properly and delays taking more than the usual hour [35]. However, the authors provided little detail as to why the number of e-faxes received, and the timeframe of nine weeks post-implementation was selected as the outcome measure for assessing the effectiveness of their intervention. As such, their purported findings must be interpreted with circumspection, given the limitations of the study design and likely biases present.

#### Care Effectiveness

One study examined how the use of interoperable EHRs by clinicians across inpatient and outpatient settings affected health outcomes and the follow-up care that patients received. Reed et al [34] explored how the rates and modes of follow-up for patients with diabetes changed after the incremental introduction of a new inpatient EHR system integrated with an existing outpatient EHR. The authors found a statistically significant reduction in the rates of in-person office visits (56%-50%) and outpatient laboratory testing (32%-31%) [34]. However, secure messaging and phone calls remain unchanged. Overall, the follow-up rates decreased from 73% to 69%.

#### Productivity

Of the 12 studies, 3 investigated the impact of interoperability on the efficiency of clinicians [33,41,42]. Of these, one study quantified the exact time saved by the clinicians [42]. Munck et al [15] primarily assessed the time spent by clinicians to perform medication reconciliation. They found that the time expended per patient using an EHR interoperable with patients’ historical medication records vs. a standalone EHR system was not significantly lengthened or statistically significant (5 minutes 27 seconds vs 4 minutes 15 seconds) [15].

Two studies captured the perceived time savings from health care providers [33,41]. Lee et al [33] described the introduction of a multidisciplinary hand-over information system that is interoperable with medical records and event tracking. Compared with an existing paper-based Kardex system, nurses reported a 50% time saving [33]. It should be noted, however, that these findings should be interpreted with caution given the limited details provided and the high risk of bias.

A simulation study conducted by Koldby et al [41] primarily examined whether integrating a digital dictation system into an EHR system would reduce unintended clinical incidents. While the authors expected improvements in this domain, they also hypothesized the simultaneous emergence of some unintended consequences, such as clinical documentation storage errors resulting from the novel intervention being introduced [41]. The participants reported notable improvements in their clinical workflows and time savings, as access to dictation services, transcripts, and medical records can be made via one click in the EHR itself. However, this benefit is ultimately offset by the limited functionality of the EHR system owing to the suboptimal integration of the dictation system [41]. Frequent system lockups, inability to open windows to other commonly used applications when dictations were being performed, and poor interoperability with other hospital systems (eg, retrieving data from laboratory information systems) all contribute to curtailing potential workflow benefits [41]. Furthermore, no workflow-related benefits were observed for other supporting clinical staff, such as medical secretaries [41].

#### Cost Savings

Of the 12 studies reviewed, only Biltoft et al [40] explored the cost savings made possible by using interoperable EHR systems. Their study attributed the introduction of smart pump-EHR interoperability to a reduction in lost revenues (US $980,000 vs US $610,000) [40]. Other indirect cost benefits, such as reducing documentation times by nurses resulting in annual cost savings of US$ 2,452,800 were also hypothesized [40].

### Risk of Bias Assessment

**Figure 2:**
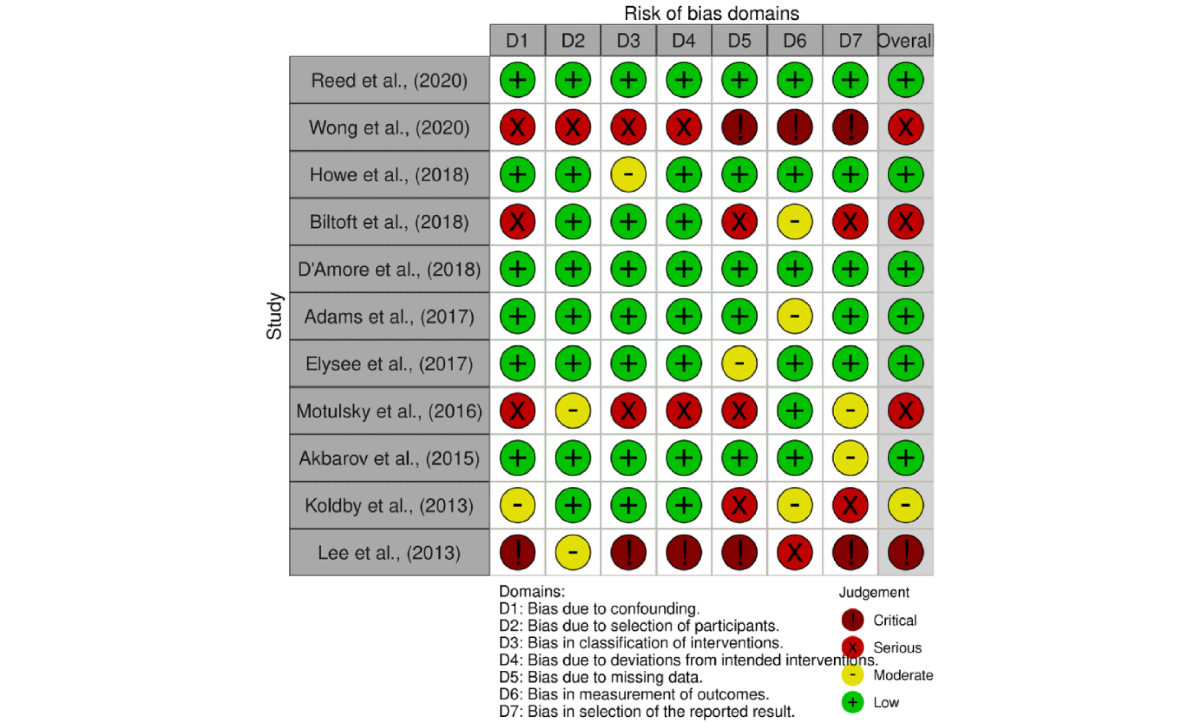
Risk of Bias in Non-Randomized Studies—of Interventions traffic lights plot for domain-level risk of bias judgments for nonrandomized studies.

**Figure 3:**
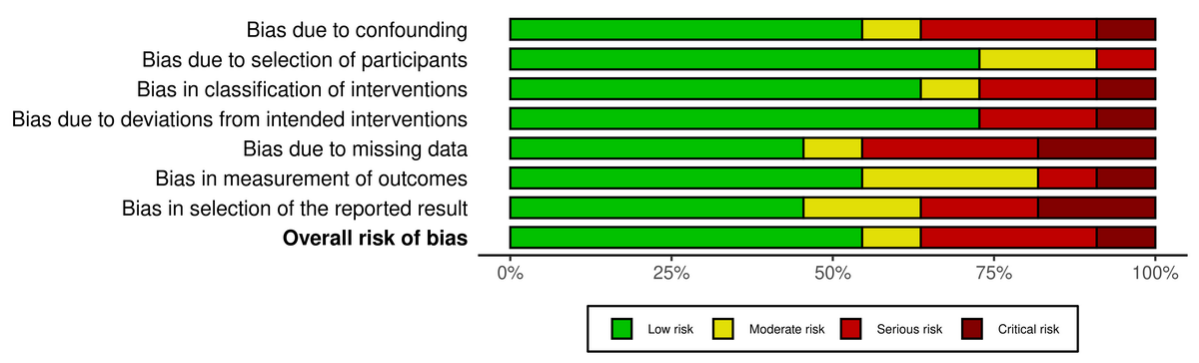
Risk of Bias in Non-Randomized Studies—of Interventions summary plot of biases present in nonrandomized studies included in the review [31-42].

**Figure 4:**
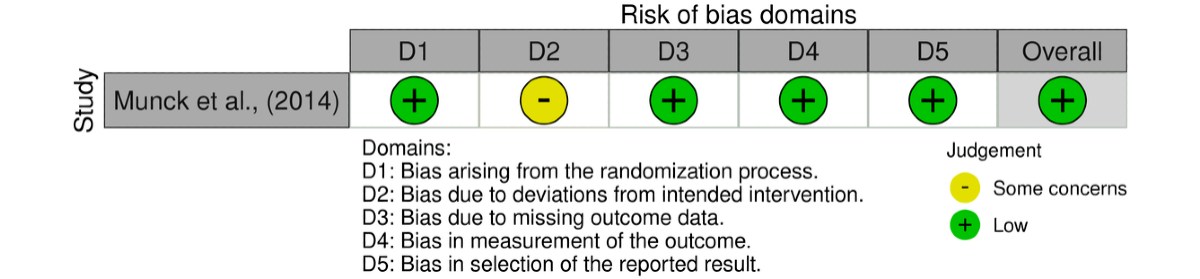
Risk of Bias 2 traffic lights plot for domain-level risk of bias judgments in randomized studies.

Upon assessing the overall risk of bias in the included studies (n=12), 7 were determined to be of low risk, 1 of moderate risk, 3 of serious risk, and 1 of critical risk (Figures 2-4). A study was considered “overall low risk” if at least 50% of the domains were rated “low risk.” Studies that had 2 or more domains rated “moderate risk” or higher were rated based on the most numerous lowest-risk domain rating. Despite the heterogeneity of previous studies, many of the studies’ data were derived from examining commonly available parameters in EHRs themselves (eg, medication lists, PSEs, or alerts) or subjective surveys of health care workers before and after changes to improve EHR interoperability were made. No studies provided evidence of a preceding registered study protocol being published. Incomplete or missing data appeared to be the most common risk of bias; many of these studies only presented their aggregated findings, rather than sharing a more detailed quantitative breakdown of their assembled data.

## Discussion

### Summary of Principal Findings

Despite similar overall aims, study objectives, and outcome measures, the studies included in this review varied widely (Multimedia Appendix 1). Most of the studies explored more than one outcome measure (n=9). The most frequently evaluated outcome measure was medication safety or reconciliation (n=7) [32,36-40,42], followed by reported PSEs (n=6) [33,34,36,38,40,41], data sharing, accuracy, and errors (n=5) [31,32,35,36,40], productivity (n=3) [33,41,42], care effectiveness (n=1) [34], and costs (n=1) [40].

Altogether, EHR interoperability appeared to have some beneficial effects on both PSEs and medication safety or reconciliation. Implementing interoperable EHRs was also associated with reduced data entry errors and improved overall data quality and scope [36,40]. However, the impact of EHR interoperability on timeliness and improvements to clinical workflow remains inconclusive; as described by Munck et al [42]*,* where the time expended to complete the clinical task increased following EHR interoperability with a community medication list.

The least used outcome measure in the reviewed studies was efficiency, with only one study explicitly mentioning changes to the cost of care associated with EHR interoperability [36]. Through the introduction of an EHR system interoperable with smart infusion pumps, participating hospitals were reported to theoretically benefit from cost savings derived from lost charges for infusions in outpatient settings and lost revenue [40].

### Strengths and Limitations

This systematic review has several strengths. It evaluated a comprehensive body of evidence published between 2010 and 2022 and retrieved 12 studies. To ensure the transparency of our search strategies, a preceding protocol paper was published, and best practice guidelines were adhered to generate these results [25]. Only studies that focused specifically on EHRs, interoperability, quality of care, and patient safety were included. Two researchers were involved in the screening and reviewing processes, with a third senior researcher to arbitrate any discrepancies that arose. The final list of included studies was determined only after a consensus was reached by all 3 researchers upon iterative consultation. Our findings were subsequently mapped onto a well-established framework for care quality commonly used in health care research [15].

However, there are notable limitations to our systematic review. The first is the decision to restrict the review to include only publications completed in English and focused on HICs. This limits the ability of this review to capture the experiences of EHRs in non-English health care settings or low- and middle-income countries. Although EHR systems are commonly found in HICs, and thus would likely have more extensive experience regarding the problem of interoperability, the authors acknowledge that these inclusion criteria can introduce a risk for bias.

Second, our study highlights the positive impact of interoperable EHRs on various outcome measures; we did not identify many negative findings. This relative lack of reported negative impact can likely be attributable to publication bias, where studies with negative findings are less likely to be published, and thus not available to be included in our review. Health IT interventions with positive outcomes also often garner more robust follow-up research efforts and deflect resources away from exploring interventions that demonstrate negative results, thus further reducing the visibility of studies investigating the latter [45].

Third, the heterogeneity of study types, contextual parameters regarding the EHR interventions examined, and study outcomes made it difficult to draw any direct comparisons between the findings of the studies. Considering this heterogeneity, we have summarized these differences in detail in Tables 1 and 2 to provide a transparent overview of the differences across studies.

Finally, the studies included in our review had an overall high risk of bias. Even more robust studies that manage to provide greater specificity in their methods and results are often limited in scope or outcomes assessed. We examined these in detail using recognized tools and provided a comprehensive summary, so that the results can be interpreted despite these limitations. Together, these limitations made it challenging to draw any definitive conclusions regarding the overall magnitude of the effect that EHR interoperability has on improving patient safety or care quality, as well as its generalizability to other health systems.

### Comparison With Prior Work

Our review findings are generally in line with those of previously published systematic reviews. These earlier reviews tended to focus on the initial adoption of EHRs in acute health care settings [46,47]. As EHR availability matured, more recent reviews began to consider EHRs’ relationships with other topics such as interoperability, examining how it affects outcome measures in diverse settings [45]. No systematic review has specifically investigated the relationship between interoperability and patient safety or quality of care.

A systematic review by Chaudhry et al [46] explored the effects of HIT adoption on quality of care and efficiency [46]. This study concluded that the introduction of HITs resulted in greater adherence to guidelines or protocols and had a positive effect on improving medication safety (ie, higher rates of adverse drug event identification and reduction in adverse drug events) [46]. Chaudhry et al [46] also described some negative consequences of their adoption and use, including mixed effects on health care provider time use and a general lack of reliable data to determine financial costs. The authors emphasized the urgent need for research into commercial EHR systems and the adoption of common standards for use in HIT research [46].

A systematic review by Jones et al [47] examined how HIT implementation affected the functionalities (ie, health care quality, safety, and efficiency) described in the meaningful use incentive program in the United States. Of the 236 articles published between 2010 and 2012 included in the review, 170 investigated care quality outcomes, 46 explored the effects of HIT on patient safety, and 62 pertained to efficiency outcomes [47]. Most studies (78%) found that HITs had a positive impact on improving patient safety; however, several papers highlighted that some aspects, such as alert fatigue, could have a negative impact on medication safety and clinical workflows [47].

Although the review by Jones et al [47] review covered HITs instead of solely EHRs, the authors described a similar impression of the HIT literature landscape as that observed in our study: diverse outcome measures, unreliable reporting of findings, and inconsistent study quality, resulting in an unclear understanding of the impact on the quality of care that patients received.

Another noteworthy review was that by Rahurkar et al [48]*,* who examined how HIE use affected health care measures such as costs, use of services, and care quality. The review included 27 articles, with the majority focusing on HIE use in the United States (70%), emergency department settings (52%), and hospitals (26%) [48]. The authors noted that many observational studies reported HIEs as having a positive effect on their outcome measure of interest, with a large proportion of those reporting on quality of care (80%). However, when accounting for study design, especially those using methods with higher internal validity (eg, randomized control trials), the authors found no strong evidence supporting HIE use being causally related to any purported benefits [48].

The review by Reis et al [3] was perhaps the closest attempt to examine how EHR interoperability may affect the 6 facets of care quality. The authors found that eHealth systems with information exchange capabilities could potentially help with the automated detection of health care-acquired infections or patient harm and work efficiency, although benefits such as enhancing documentation accuracy and quality are less clear [3]. The authors identified no studies that investigated the potential cost benefits of interoperability [3]. Although only a limited number of papers met the inclusion criteria and examined an assortment of eHealth systems used, the authors concluded that eHealth systems with interoperability have some positive effects on patient care quality in certain clinical applications (eg, disease, event surveillance) [3].

### Implications for Policy and Further Research

Realizing the well-recognized benefits of interoperable EHRs is often hampered by a multitude of contextual factors. Contributing factors such as the considerable expense associated with introducing new health IT systems and infrastructure, differing procurement policies between health systems, lack of business incentives, and fundamental technical challenges impede the introduction of interventions aimed at addressing interoperability between EHRs [23,49,50].

As demonstrated in the literature, piecemeal interventions aimed at linking EHR systems at a technical level may alleviate bottlenecks in one area of the clinical pathway but only to have the gains undermined elsewhere and culminate in not meaningfully improving the patient’s care during their clinical encounter.

Given that interoperable EHR use by health care workers has already been extensively researched, similar efforts must be devoted to investigating the exact benefits to patient safety or quality of care from the perspective of patients and caregivers. In addition, further research efforts should be devoted to exploring EHR interoperability interventions that do not yield positive results. Understanding these failed interventions and their underlying causes can help better inform our insight into interventions that have shown greater promise.

The adoption of common technical standards and support for regulatory and legislative alignment will prove valuable in further ushering in greater interoperability in the coming years. Likewise, unifying a common set of key performance indicators is also needed to allow for more transparent and comparable metrics for the continued monitoring and evaluation of new policies. Outcomes such as PSEs, medication reconciliation, and time savings may be obvious starting points, but a more consolidated list of universal outcome measures is essential to accurately quantify the effect of these complex interventions.

### Conclusions

Our systematic review found that interoperable EHRs have had a positive impact on certain aspects of patient safety, such as medication reconciliation, reducing PSEs, lowering the risk of data errors, and improving data quality. However, a reliable determination of their true measure of effect with the available assortment of evidence remains difficult. Current evidence underscores the value and importance of continuing to implement greater interoperability in the upgrading of existing EHR systems and the procurement of new ones. In practice, however, relying on present findings to inform exact outcome measure improvements to expect, may prove challenging.

As clinical data increase both in volume and complexity, EHR interoperability will become indispensable for realizing a more streamlined and sustainable workflow for clinicians. Standardizing outcome measures, examining EHR interoperability through the lens of systems complexity, and greater inclusivity of patient perspectives in EHR-related research will be necessary to better evaluate the growing importance of interoperable EHRs in high-income health care settings in the foreseeable future.

## Appendix

Multimedia Appendix 1 Main outcomes table.

Multimedia Appendix 2 PRISMA (Preferred Reporting Items for Systematic Reviews and Meta-Analyses) checklist.

## Abbreviations

EHR: electronic health record
HIC: high-income country
HIE: health information exchange
HIT: health information technology
IoM: Institute of Medicine
PICO: population, intervention, control, and outcomes
PRISMA: Preferred Reporting Items for Systematic Reviews and Meta-Analyses
PSE: patient safety event
