# Supplementary material for: The Impact of Electronic Health Record Interoperability on Safety and Quality of Care in High-Income Countries: Systematic Review
Source: J Med Internet Res. 2022 Sep 15;24(9):e38144. doi: 10.2196/38144 (PMC9523524; doi:10.2196/38144)
Supplement: Multimedia Appendix 1 [file jmir_v24i9e38144_app1.docx]

| Author(s) | Publication Year | Intervention(s) | Outcome(s) | Risk of Bias Judgement |
| --- | --- | --- | --- | --- |
| *M. Reed et al.,* | 2020 | - Implementation of an inpatient EHR which integrates with a pre-existing outpatient EHR system across 17 hospitals | - Follow-up rates - Within 7 days post-discharge, follow-up rates decreased from 72.8% to 69.2%. - Within 30 days post-discharge, follow-up rates decreased from 94.8% to 94.1%. - Modality of follow-up - Within 7 days post-discharge, telemedicine and laboratory tests without the need for in-person office visits increased from 22.9% to 27%. - In-person office visits decreased from 55.9% to 50.5%. - Laboratory testing decreased from 32% to 30.7%. - No statistically significant changes were noted for rates of telephone visits of secure messages. - Adverse clinical events post-discharge - 30 days post-discharge saw no statistically significant changes to rates of emergency department visits (16.7% vs. 16.4%) or readmissions (9.5% vs. 9.4%). | 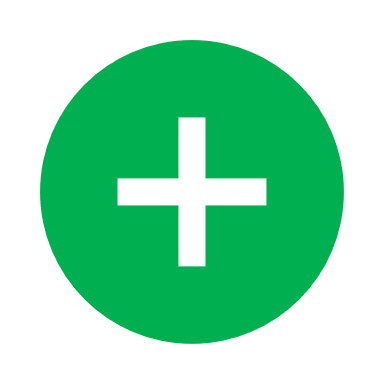 |
| *S. Wong et al.,* | 2020 | - Introduction of software to transform outpatient fax input into data fully integrated into inpatient EHRs. | - 9 weeks post-implementation of e-faxes integration with inpatient EHRs, the intervention was recorded to be utilised 287 times across the three-hospital health network. - User feedback on the new process was largely positive. However, shortcomings such as missing pages of documents or delays of over one hour in their integration with the EHRs were reported. | 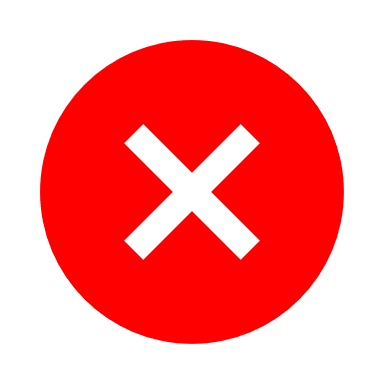 |
| *J. Howe et al.,* | 2018 | - Review of free-text patient safety event reports from the Pennsylvania Patient Safety Authority database from 2013-2016. | - Of 1.735 million safety events, 1956 (0.11%) are related to EHRs; 557 (0.03%) may have contributed to actual patient harm. - Interoperability accounted for 102 events (18%): 42 of which were related to reviewing of results and 38 to order placement. - Availability of information accounted for 50 events (9%):25 of which were related to order placement and 13 to medication administration. | 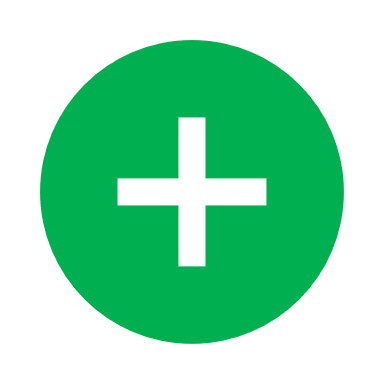 |
| *J. Biltoft et al.,* | 2018 | - Introduction of infusion smart pumps interoperable with EHRs to allow for automatic pre-population of infusion parameters. | - Medication safety   - Improvements in the reduction of the average number of keystrokes to be entered to program an infusion by 86% (15 to 2). This equates to negating 3.5 million keystrokes/opportunities for error across eight participating hospitals, in a month.   - Rate of appropriate entry of patient identification information by pump users increased from 35.5% to 81.0%.   - Average number of pump alerts decreased 22% (1,845 to 1,447) monthly. Average number of infusions requiring to be reprogrammed due to an alert dropped 19% (119-96)   - Annual self-reported safety events dropped from four to one. - Compliance to newly implemented integrated EHR technologies was high, averaging 70-80% during the initial seven months, increasing to 90% in more enthusiastic teams. - Reduced lost charges for outpatient infusions by approximately 40% (11.9%-7.4%), $370,000 in savings. | 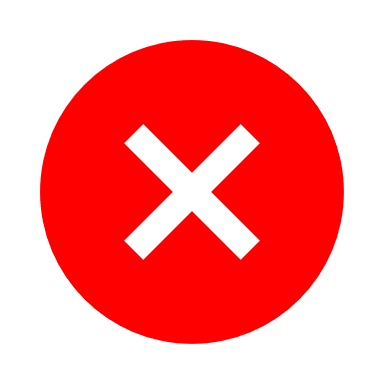 |
| *J. D'Amore et al.,* | 2018 | - EHRs certified to the new Consolidated Clinical Document Architecture (C-CDA) 2.1 documentation standard | - Four examples of interoperability affecting data exchange with clinical relevance were highlighted:   - Coded drug allergies not matching their description   - Inaccurate records of patient parameters using incorrect units   - Inaccurate medication dosages, impacting medication administration and reconciliation   - Omission of code/units used for laboratory results. - Overall, authors noted ‘notable improvements’ in the quality of C-CDA documents attributable to ‘modest improvements’ in interoperability.   - Breadth of clinical information included in C-CDA documentation has increased   - Plans for treatment are now better structured. Number of schematron errors, key patient safety/data quality issues have decreased compared to previous evaluations.   - Appreciable general improvement in the knowledge surrounding the use of C-CDA documentation standards in the industry in the last few years. | 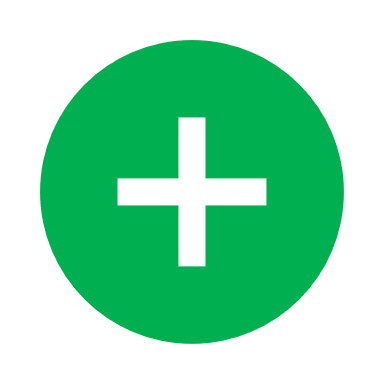 |
| *K. Adams et al.,* | 2017 | - Review of patient safety event reports documenting safety hazards related with interoperability between EHRs and other health IT. Reports derived from the Pennsylvania Patient Safety Authority’s Pennsylvania Patient Safety Reporting System and a large healthcare system in the Mid-Atlantic United States between 2009-2016. | - 209 patient safety events out of 2,625 (8%), were attributable with EHR interoperability issues.   - 60 (29%) pertained to medication-related events, 55 (26%) laboratory, 43 (21%) radiology, 22 (11%) device-related, and 29 (14%) ‘other’. - Patient harm scores for the 209 safety events were analyzed   - Most interoperability-related safety events did reach patients, but most did not cause any harm.   - 38 (18%) rated as unsafe conditions, 55 (26%) did not reach patients to cause harm, 111 (53%) did reach patients but did not cause harm, and 5 (2%) events did reach patients to cause harm.     - A majority of safety events pertaining to medications (70%), laboratory tests (60%), and radiology (51%) did reach patients. Device and other interoperability related category safety events were usually caught before reaching patients to cause harm.     - Majority of safety events resulting for interoperability issues are related to issues receiving information from other EHR systems WITHIN an organization rather than sending information externally. | 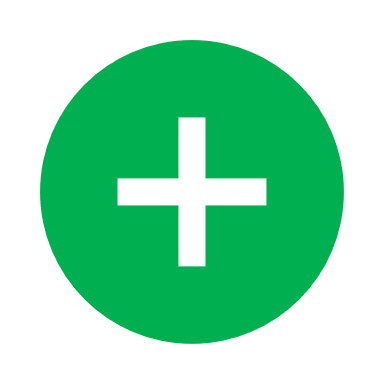 |
| *G. Elysee et al.,* | 2017 | - Partial least squares-structural equation modeling technique applied to variables obtained from the 2013 American Hospital Association annual survey Information Technology (IT) supplement to assess relationship between EHRs, interoperability, and medication reconciliation | - The relationships between hospitals’ adoption of electronic health information exchange, interoperability, and medication reconciliation capabilities are significant, positive, and cyclic. If any one of them decreases, then the others will decrease as well. - Building blocks of electronic health information exchange capability factor consisted of variables representing health IT functionalities that enable exchange, not only between unaffiliated hospitals and ambulatory facilities, but also with patients. - Interoperability is related to hospitals’ ability to produce electronic transition of care summary documents that are interoperable, in that they must conform to the structure and vocabulary standards specified by Health Level Seven (HL7) Continuity of Care Documents. | 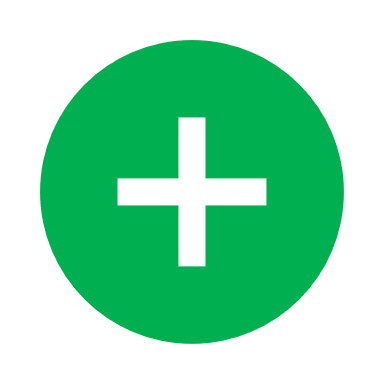 |
| *A. Motulsky et al.,* | 2016 | - Implementation of SQIM electronic application to allow clinicians to access both current and past medication lists for all patients in Quebec province. | - Two-thirds (63.9%) of patients had inaccurate medication lists generated from the SQIM. - Three types of discrepancy were observed:   - 44.6% occurred when a medication was listed as current in the SQIM but should not have been (false positive)   - 43.9% when a medication was not listed as current in the SQIM but should have been (false negative)   - 11.5% were duplicate medications in the SQIM.   - 67.2% of the discrepancies were related to an inconsistency between the practices in community pharmacies and the design of the application (e.g., putting new prescriptions on hold when dispensing an old prescription for the same medication); while 21.3% were related to the rule for attribution of the “current” flag in the SQIM. | 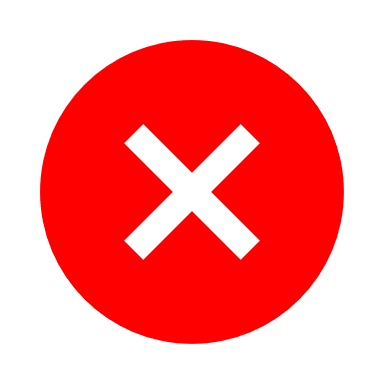 |
| *A. Akbarov et al.,* | 2015 | - Integrated hospital-community shared patient records (Salford Integrated Records SIR) at Salford Royal NHS Foundation Trust | - Polypharmacy and higher age groups were associated with increased odds of prescription safety risks, but lower odds of medication monitoring failures. - Odds of missing medication monitoring event for males are 25% lower than for females. - Odds of missing medication monitoring events was 50% for patients served by training practices than non-training practices. - Practices in deprived areas were at 3x higher risk of to miss medication monitoring. - Higher number of monitoring indicators reported, though unclear if this is due to the large number of secondary care data and requests originating from primary care being misreported or patients visiting secondary healthcare settings directly. | 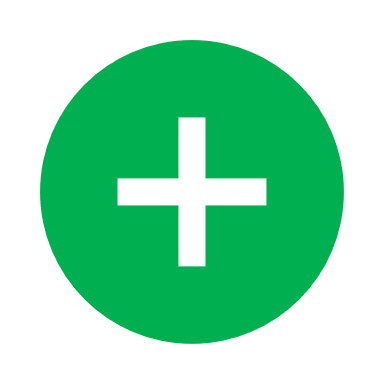 |
| *L. Munck et al.,* | 2014 | - EHRs interoperable with Shared Medication Record (SMR), a shared national service containing active medications and past prescriptions for all Danish citizens vs. EHRs without SMR, in the emergency department setting | - A total of 62 consecutive consultations were observed (28 EMR, 34 SMR); 18 physicians participated in the study.   - No statistical difference in the median time expenditure for medication reconciliation and median time to complete consultations. This was consistent regardless of the physician’s level of clinical experience.   - Based on questionnaire feedback from physicians, SMR integration was deemed feasible and does not interrupt the routine workflow.   - Unanimous consensus that physicians in the emergency department should have access to SMR data.   - Medication reconciliation process encouraged greater patient empowerment and engagement with their healthcare provider.     - Patients have expectations that healthcare providers use SMRs for medication reconciliation processes, but also have concerns regarding privacy and security. | 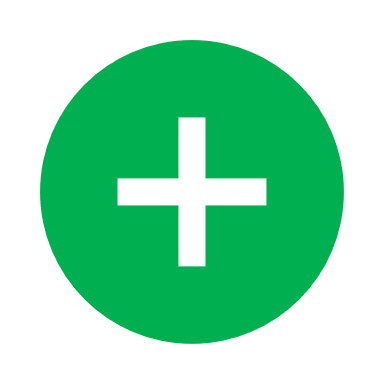 |
| *S. Koldby et al.,* | 2013 | - Integration of clinical information system (CIS) in the form of a new digital dictation service, into existing EHR systems to assess its impact on clinician workflow and clinical consequences | - Clinical simulation test showed that EHRs freeze during use of integrated digital dictation.   - This has potential implications on patient safety as one cannot use laboratory information systems and limits situational awareness regarding the patient.   - This is also detrimental to doctor’s workflows and was not integrated in a way which is inclusive of the workflows of other medical professionals (e.g., medical secretaries) - Integrated dictation was deemed to be useful, time-saving, convenient, and easy to learn to use. - The simulations showed that the integration between digital dictation and the EHR is easy to use. It supported the workflow during dictation, but not the entire workflow with common use of other applications. | 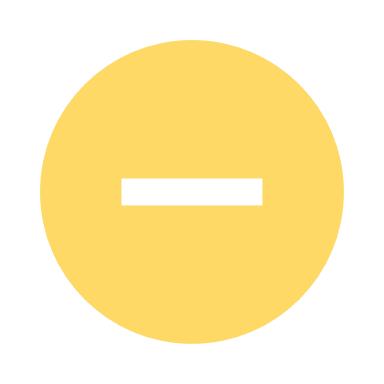 |
| *Y. Lee et al.,* | 2013 | - Implementation of a new cross-disciplinary team multidisciplinary handover information system integrated with medical record browsing, and event tracking vs. existing Kardex system | - Implementation saved 50% of the time for writing the Kardex for nurses. - Handover accuracy rated at 100% three months after implementation of the new multidisciplinary handover system. No significant changes to adverse events (no details provided). | 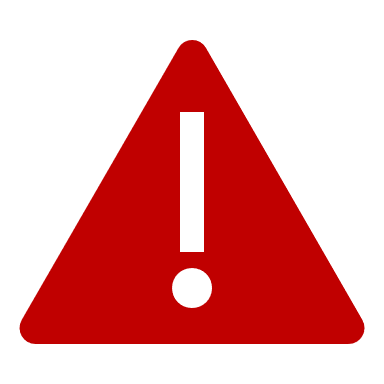 |

**ROBINS-I Risk of Bias Judgement**

| 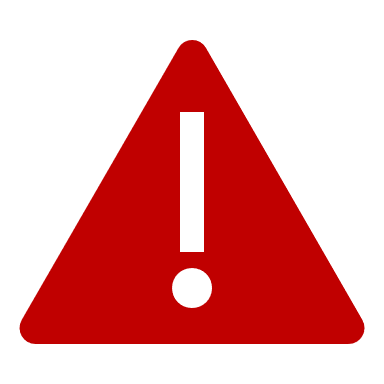 | Critical |
| --- | --- |
| 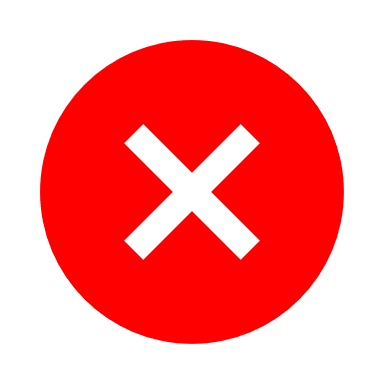 | Serious |
| 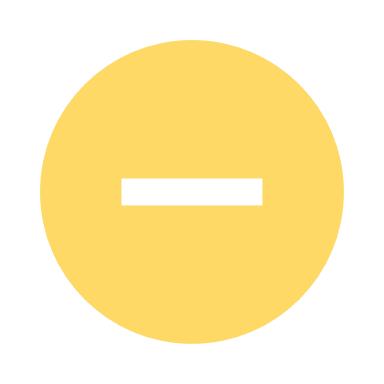 | Moderate |
| 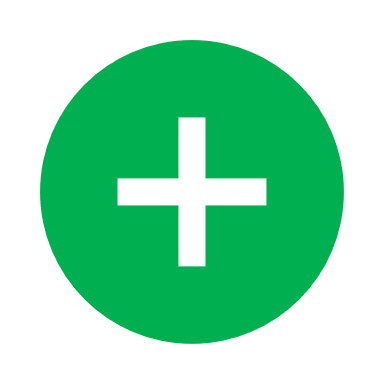 | Low |
